# Brazilian Journal of Otorhinolaryngology - A landmark

**DOI:** 10.5935/1808-8694.20130072

**Published:** 2015-10-08

**Authors:** João Ferreira de Mello

**Affiliations:** Senior Associate Professor at the Medical School of the University of São Paulo. Head of the Allergy Group in ENT - University Hospital of the Medical School of the University of São Paulo. Editor-in-Chief of the Brazilian Journal of Otorhinolaryngology 2005-2010.

Eight years ago I was invited to be the editor-in-chief of the Brazilian Journal of Otorhinolaryngology - which had just been indexed in Medline. The invitation was a surprise; however, with the support from the director of publications, Prof. Silvio Caldas Neto, the deputy director of publications, Prof. Regina Helena G. Martins and the Chairman of the ABORL-CCF, Prof. Richard Voegels, I accepted to face the challenges imposed on me.

Besides the scientific aspect, paper submission and the review by two reviewers had just migrated to the “virtual world”. We had many papers awaiting review and acceptance for publication. Structurally, the journal is formatted with a limited number of original papers, case reports and review papers, so unfortunately it was not possible to publish them quickly.

Some of the authors, when meeting me at random would ask: “João, when will my paper be published?”; “It has been a while that it was accepted and has not yet been published.”; “My study will be outdated.” These were some of the things I heard. I think they were all right in wanting their papers, clinical trials, case reports and observations to be published as soon as possible, however, we had a physical limitation. I chose to use the order of the paper submission protocol number as criteria for setting the publishing queue. Some colleagues would not agree with this for fear that their papers would become obsolete; however, I saw this as the most honest approach with all the authors. At the time, our journal was one of the major national publications in its field, and it still is. Thus, it serves as a tool to spread the knowledge created in Brazil for all our members.

Because we acquired a high profile due to our presence in MedLine, many foreign authors began to submit papers for publication in our journal. This was another big step conquered representing its internationalization.

I am very proud to have participated as editor-in-chief of our journal over the administrations of Prof. Richard Voegels and that of Prof. Ricardo Ferreira Bento. Likewise, I am grateful to the associate editors and reviewers with whom I had the opportunity to work. At the end of five years, we had faced and overcome many challenges and setbacks, managing to maintain the high scientific level of our journal, significantly expediting the publication of papers and taking the first steps to be accepted by the ISI, which occurred under Prof. Wilma Terezinha Anselmo-Lima, whom I congratulate for the competence and the work she has been doing for our journal.

This year, the Brazilian Journal of Otorhinolaryngology turns 80. It is not the journal that deserves congratulations, but rather all colleagues who directly or indirectly contributed to its development and establishment as the most important scientific journal of the Brazilian Association of Otolaryngology and Neck and Facial Surgery.

